# A retrospective analysis of bone mineral status in patients requiring spinal surgery

**DOI:** 10.1186/s12891-018-1970-5

**Published:** 2018-02-13

**Authors:** Tobias Schmidt, Katharina Ebert, Tim Rolvien, Nicola Oehler, Jens Lohmann, Luca Papavero, Ralph Kothe, Michael Amling, Florian Barvencik, Haider Mussawy

**Affiliations:** 1 Department of Osteology and Biomechanics, University Medical Center Hamburg-Eppendorf, Lottestraße 59, 22529 Hamburg, Germany; 2 Department of Orthopedic Surgery, University Medical Center Hamburg-Eppendorf, Martinistrasse 52, 20246 Hamburg, Germany; 3 Clinic for Spinal Surgery, Schoen Klinik Eilbek, Denhaide 120, 22081 Hamburg, Germany

**Keywords:** Osteoporosis, Vitamin D deficiency, Secondary hyperparathyroidism, Hypochlorhydria, Anti-osteoporotic medication

## Abstract

**Background:**

Impaired bone quality is associated with poor outcome of spinal surgery. The aim of the study was to assess the bone mineral status of patients scheduled to undergo spinal surgery and to report frequencies of bone mineral disorders.

**Methods:**

We retrospectively analyzed the bone mineral status of 144 patients requiring spinal surgery including bone mineral density by dual-energy X-ray absorptiometry (DXA) as well as laboratory data with serum levels of 25-hydroxyvitamin D (25-OH-D), parathyroid hormone, calcium, bone specific alkaline phosphate, osteocalcin, and gastrin. High-resolution peripheral quantitative computed tomography (HR-pQCT) was additionally performed in a subgroup of 67 patients with T-Score below − 1.5 or history of vertebral fracture.

**Results:**

Among 144 patients, 126 patients (87.5%) were older than 60 years. Mean age was 70.1 years. 42 patients (29.1%) had suffered from a vertebral compression fracture. 12 previously undiagnosed vertebral deformities were detected in 12 patients by vertebral fracture assessment (VFA). Osteoporosis was present in 39 patients (27.1%) and osteopenia in 63 patients (43.8%). Only 16 patients (11.1%) had received anti-osteoporotic therapy, while 54 patients (37.5%) had an indication for specific anti-osteoporotic therapy but had not received it yet. The majority of patients had inadequate vitamin D status (73.6%) and 34.7% of patients showed secondary hyperparathyroidism as a sign for a significant disturbed calcium homeostasis. In a subgroup of 67 patients, severe vertebral deformities were associated with stronger deficits in bone microarchitecture at the distal radius compared to the distal tibia.

**Conclusions:**

This study shows that bone metabolism disorders are highly prevalent in elderly patients scheduled for spinal surgery. Vertebral deformities are associated with a predominant deterioration of bone microstructure at the distal radius. As impaired bone quality can compromise surgical outcome, we strongly recommend an evaluation of bone mineral status prior to operation and anti-osteoporotic therapy if necessary.

## Background

Osteoporosis is a progressive disease that is characterized by a decrease in bone mass density and structures changes in trabecular and cortical bone leading to an increased risk of fractures. It is estimated that over 200 million people worldwide suffer from osteoporosis [1]. Moreover, it has been predicted that the lifetime risk of osteoporotic fractures in the United States is approximately 30–50% in women and 15–30% in men and that more than 40% of postmenopausal women will suffer one or more fragility fractures in their remaining lifetime [2].

Vertebral compression fractures are the most common complication of osteoporosis and strongly affect patients’ overall health. The presence of a vertebral fracture increases the risk of a new vertebral fracture by five-fold [3] and patients with vertebral fractures often suffer from severe pain that can lead to long-term care and hospitalization which is associated with further complications [4]. Furthermore, patients with vertebral fractures are at much greater risk for developing changes in spine structure as kyphotic deformities and spinal stenosis due to sagittal imbalance and degenerative changes [5].

Osteoporosis can be diagnosed by low bone density measured by dual-energy X-ray absorptiometry (DXA) or by a fragility fracture. Although DXA is the gold standard to measure areal bone mineral density (aBMD), it cannot be used to gain insights into three-dimensional bone architecture changes or distinguish between the cortical and trabecular compartments. In addition, it is well known that fractures often occur in patients with a T-score above − 2.5, who therefore do not meet the World Health Organization (WHO) criteria of osteoporosis [6]. These limitations of DXA are important because patients with vertebral fractures not only often have degenerative bone changes that influence the correct interpretation of the lumbar BMD, but in such cases DXA-measured aBMD at the lumbar spine may yield false high values.

In patients with vertebral fractures, the vertebral bodies are characterized by reduced bone volume tissue and the loss of trabeculae [7]. Interestingly, similar deficits are also observed in peripheral bone [8, 9]. This could explain why these patients are at particular risk of vertebral and peripheral fractures in the future [10, 11]. It also suggests that one way to improve the diagnosis of vertebral fractures is by assessing the peripheral bone architecture via high-resolution peripheral quantitative computed tomography (HR-pQCT) [12]. Indeed, several studies show that HR-pQCT measurements of the peripheral bone microstructure are predictive of osteoporosis-related fractures [13, 14].

In the recent years there have been significant advances in the management of osteoporosis. Many studies have investigated the effect of vitamin D and calcium supplements on osteoporosis and fracture risk [15]. Low circulating levels of 25-hydroxyvitamin D (25-OH-D) may lead to increased secretion of parathyroid hormone (PTH) which in turn induces bone loss through increased bone resorption. Despite the importance of vitamin D for maintaining balanced calcium homeostasis, deficiency is highly prevalent in Europe population particularly in northern Europe countries like Germany, where in contrast to Scandinavia food fortification with vitamin D is still lacking [16, 17].

In this study, we collected data of bone mineral status and bone quality in patients scheduled for spine surgery and report rates of untreated osteoporosis, vitamin D deficiency and hyperparathyroidism in a patient cohort in northern Germany.

## Methods

### Study group

Since early 2015 to the end of 2016 a total of 144 consecutive adult patients (> 50 years of age), who were scheduled for spine surgery in a single center in north Germany, were examined at our institution and included in this retrospective cross-sectional study. All patients were interviewed for previous fractures, medical treatment including anti-osteoporotic treatment and vitamin D supplementation, and associated diseases. Patients with diabetes mellitus type 1, treatment with glucocorticoids lasting over 3 months or tumors were excluded. Patients whose vertebral fractures were the result of major trauma were also excluded. Indication for specific anti-osteoporotic treatment was determined by using the evidence-based (S3) guidelines of German Association of Osteology (DVO). This guideline currently recommends the use of denosumab, raloxifene, bazedoxifene, estrogens**,** alendronate**,** risedronate, ibandronate, zoledronic acid, teriparatide and strontium ranelate as specific anti-osteoporotic treatment medication. Informed consent was obtained from all patients for the retrospective and anonymized database studies. This study was performed in accordance with the Declaration of Helsinki. The local ethics committee of the University Medical Center Hamburg-Eppendorf approved this retrospective study (PV5271).

### Dual-energy X-ray absorptiometry (DXA)

**Fig. 1:**
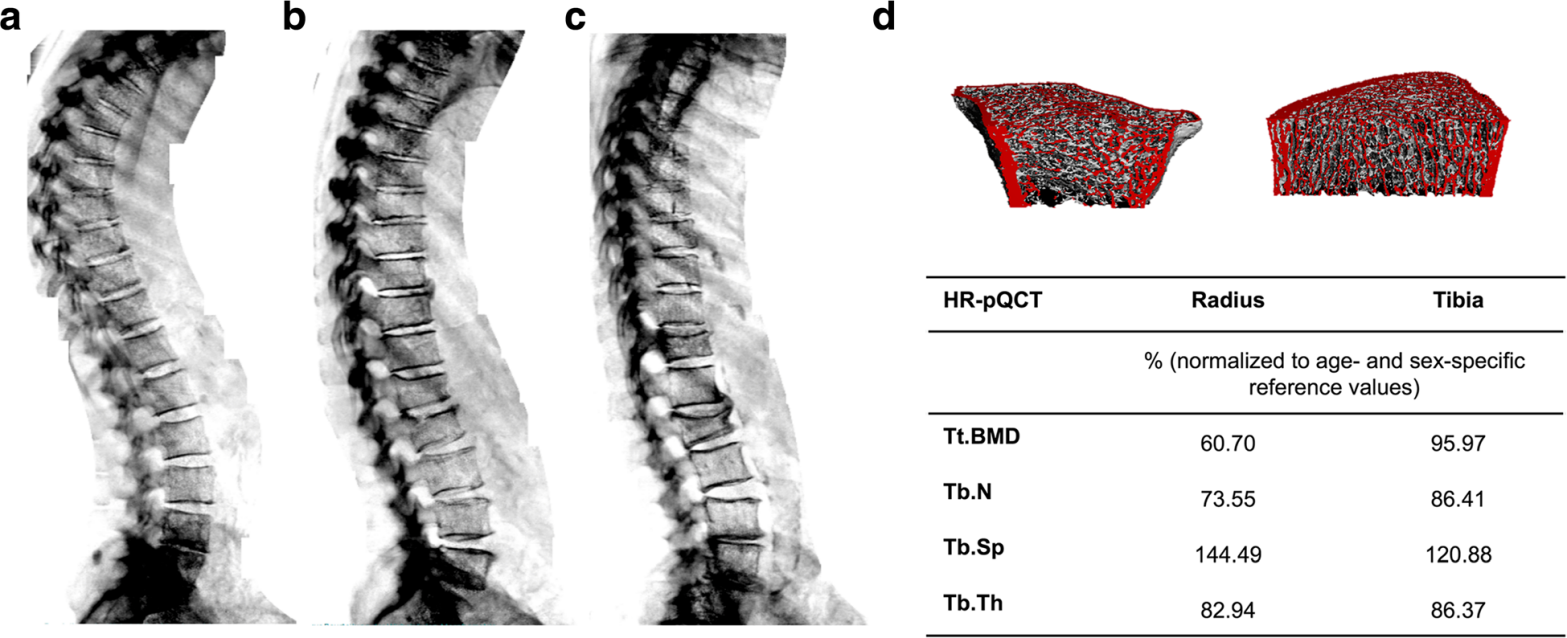
**a** Sample images of lateral vertebral fracture assessment by DXA in patients without vertebral fracture; **b** with moderate deformity of the first and second lumbar vertebrae; **c** and with severe deformity of the second lumbar vertebra and moderate deformity of the twelfth thoracic vertebra. **d** Sample image of the peripheral quantitative computed tomographic analysis of the distal radius and distal tibia of a patient with severe vertebral deformity. Note that the same patient exhibits a strong decrease in the trabecular compartment at the distal radius and a less pronounced decrease in trabecular variables at the distal tibia. Values are normalized to age-, site-, and sex-specific reference values. *Abbreviations*: HR-pQCT, peripheral quantitative computed tomography; Tt.BMD, total volumetric bone mineral density. Tb.N; trabecular numbers Tb.Sp; trabecular separation Tb.Th; trabecular thickness

All patients underwent aBMD measurements by DXA (iDXA, GE Healthcare, UK). Three skeletal areas, the both proximal femur and the lumbar spine (L1–L4), were measured by DXA. The patients were placed in the supine position and scanned according to the manual supplied by the manufacturer. The detected aBMD of the projected bone area was expressed in grams per square centimeter (g/cm2), and the corresponding T-, and Z-Score was calculated using the reference database provided by the manufacturer. DXA measurement at lumbar spine had to be excluded due to degenerative osteoproliferative changes in 25 patients and DXA measurement at proximal femur was not possible due to bilateral hip replacement in 13 patients. Yet, all patient in this study had at least one representative DXA measurement. The vertebral fractures were confirmed with vertebral fracture assessment (VFA) by DXA (Fig. 1a-c). Vertebral deformity severity was measured using VFA by DXA followed by grading of the vertebrae according to the semiquantitative method of Genant. Moderate deformity was defined as reduction of 25–40% in anterior, middle, and/or posterior vertebral height, while severe deformity was defined as a reduction in any of these heights by more than 40% [18].

### High-resolution peripheral quantitative computed tomography (HR-pQCT)

The patients (*n* = 67) with a T-score below − 1.5 or a history of a vertebral fracture received additional assessment of peripheral bone structure by HR-pQCT (XtremeCT®, Scanco Medical AG, Brüttisellen). HR-pQCT scans of the non-dominant distal radius (in cases of previous fracture, the contralateral limb was scanned) and the distal tibia were analyzed. The measured region was manually defined by a trained operator by placing a reference line at the endplate of the radius and tibia on a preliminary performed scout view. The same operator generated semiautomatic contours around the periosteal surface and the entire volume of interest is thereafter automatically separated into a cortical and trabecular region. A quality scan for calibration of the CT system was performed each day by using a phantom provided by the manufacturer. The scanning settings used were 60 kV/40 keV at a current of 900 μA. Each image comprised 110 slices with an isotropic voxel size of 82 μm. The following variables were analyzed by using the HR-pQCT system: total, cortical and trabecular area, total, cortical and trabecular volumetric BMD, cortical thickness, trabecular number, trabecular thickness, and trabecular separation. HR-pQCT values were normalized to age-, site-, and sex-specific reference values [19] and are expressed as percentage of corresponding reference value.

### Laboratory values

Biochemical analyses of bone metabolism markers including serum levels of alkaline phosphatase (ALP), bone specific alkaline phosphatase (BAP), 25-OH-D, calcium, parathyroid hormone (PTH) and urinary level of deoxypyridinoline (DPD) were assessed by routine lab tests for evaluating osteoporosis. Reference values were adapted from the local laboratory for each parameter. Vitamin D inadequacy was defined as a 25-OH-D level below 30 ng/ml and deficiency as levels less than 20 ng/ml. These values reflect those of widely accepted thresholds [20]. Calcium, gastrin, 25-OH-D and parathyroid hormone were measured in all patients, bone specific AP were only measured in 115 patients, osteocalcin in 111 patients and urinary levels of DPD in 107 patient.

### Statistics

The IBM SPSS statistics 22 program was used for statistical analyses. Data are expressed as mean values ± SD. Normal distribution of the data was tested with the Kolmogorov–Smirnov test. To test the differences between the study groups, we used the unpaired two-sided t-test and one-way ANOVA and post hoc Bonferroni test on the normally distributed data and the Mann–Whitney U test and Kruskal Wallis test for non-normally distributed data. *P* values of < 0.05 were considered as statistically significant.

## Results

### Study population characteristics

A total of 144 consecutive adult patients older than 50 years were included in this retrospective study. All patients were scheduled for spine surgery. The mean age and BMI was 70.1 ± 8.1 years and 26.2 ± 4.87 kg/m^2^, respectively. Among 144 patients, 96 (66.4%) were females. 42 patients had suffered from a vertebral compression fracture in the past. Almost half of the patients (48.6%) had a diagnosis of lumbar spinal stenosis. Table 1 summarizes the characteristics of the study population.

**Table 1:** Demographic and laboratory data of of patients requiring spinal surgery

	Mean (± SD) or n (%)
n	144
Sex (no. of female, %)	96 (66.4)
Mean age in yr	70.1 (8.1)
Age (no. of patients)	
50–60 yr	18 (12.5%)
60–70 yr	55 (38.2%)
> 70 yr	71 (49.3%)
Weight (kg)	74.8 (14.4)
Height (cm)	168.0 (9.4)
Height change (cm)	3.7 (2.9)
Mean BMI (± SD)	26.2 (4.7)
History of a vertebral fracture	42 (29.1%)
Unknown vertebral deformity (Diagnosed by VFA)	12 (8.3%)
Proton pump inhibitor use	44 (30.6%)
Laboratory values	
25-OH-D (ng/ml)	24.3 (11.8)
Parathyroid hormone (ng/l)	84.3 (47.1)
Calcium (mmol/l)	2.23 (0.1)
Bone specific AP (μg/l) (*n* = 115)	13.6 (7.9)
Osteocalcin (μg/l) (*n* = 111)	18.9 (11.8)
Urine DPD (*n* = 107)	6.7 (2.8)
Elevated DPD	74 (69.2%)
Elevated Gastrin	20 (13.9%)

### Bone densitometry

DXA was performed in all patients. However, DXA measurement in 25 patients at lumbar spine had to be excluded due to degenerative osteoproliferative changes. In 13 patients DXA measurement at the proximal femur was not possible due to bilateral hip replacement. Yet, all patient in this study had at least one representative DXA measurement. Osteoporosis at the lumbar spine was diagnosed in 27 of 119 patient (22.7%) whereas 22 of 131 patients (16.8%) showed T-scores below − 2.5 at the femur. Incidence of osteopenia was 34.5% at the lumbar spine and 45.8% at the femur. Table 2 shows the indication for spinal surgery and the corresponding T- and Z-scores for each subgroup. Patients requiring spinal surgery due to a compression fracture showed the lowest T- and Z-scores at the lumbar spine and at the femoral neck.

**Table 2:** T-scores and Z-scores of the DXA measurements in patients with different indication for spinal surgery (mean ± SD)

	n	T-Score lumbar spine	Z-Score lumbar spine	T-Score femoral neck	Z-Score femoral neck
All patients	144	−1.23 (1.4)	- 0.1 (1.5)	- 1.4 (1.0)	- 0.08 (1.1)
Indication for spinal surgery					
Lumbar spinal stenosis	70	−1.29 (1.73)	0.07 (1.56)	−1.62(1.15)	−0.04 (1.08)
Degenerative spondylolisthesis	35	−1.14 (1.45)	− 0.20 (1.38)	− 1.16 (0.98)	0.12 (1.10)
Herniation of lumbar disc	31	−0.77 (1.21)	−0.04 (1.41)	−1.06 (0.88)	0.32 (1.38)
Compression fracture	8	−2.6 (1.3)	−1.27 (0.91)	−1.72 (1.07)	−0.13 (1.05)

### Deterioration of bone structure at the distal radius is associated with severity of vertebral fracture

Vertebral fracture assessment (VFA) of the spine revealed 12 previously unknown vertebral deformities in 12 different patients. Fig. 1a-c shows sample images of VFA by DXA in patients without vertebral deformity (a), moderate deformity (b) and severe deformity (c). In a subgroup of 67 patients with a T-score below − 1.5 or a history of fracture, we used HR-pQCT for further assessment of bone microstructure at the distal radius and distal tibia. 21 and 12 patients of this subgroup had moderate and severe vertebral fractures, respectively. The patients did not differ significantly in terms of age (mean 71.9 vs. 71.6), weight (72.7 kg vs. 78.4 kg), height (166 cm vs. 170 cm), or BMI (26.1 vs 26.9). Interestingly, when we compared patients with vertebral deformities to patients without deformities, the cases with severe vertebral deformity had significantly lower total volumetric BMD (17.75%) as well as trabecular and cortical microstructure parameters at the distal radius (Table 3). The tibial values demonstrated similar trends, albeit less pronounced and without achieving statistical significance (Table 3). Figure 1d demonstrates images of a patient with pronounced deterioration of bone microstructure at the distal radius.

**Table 3:** Differences in bone microarchitecture between the patients with moderate/severe vertebral deformity and the patients without vertebral fractures

	No Deformity	Moderate Deformity		Severe Deformity	
n	34	21		12	
Mean age, years	71	71.9		72	
HR-pQCT variables					
Radius (normalized to age- and sex-specific reference values)	%	%	Δ %	%	Δ %
Total bone area	109.8 (15.7)	113.4 (18.5)	+ 3.5	110.2 (30.7)	+ 0.35
Cortical area	88.8 (21.7)	78.3 (27.3)	−10.5	65.4 (19.5)	−23.4*
Trabecular area	110.8 (20.4)	117.2 (24.4)	+ 6.2	116.7 (36.8)	+ 5.7
Total vBMD	94.9 (20.7)	87.2 (18.9)	−7.6	76.2 (12.5)	−18.7*
Cortical vBMD	83.3 (7.0)	80.1 (8.4)	−3.1	77.7 (6.9)	−5.5
Trabecular vBMD	100.7 (20.7)	93.5 (25.7)	−7.3	79.7 (17.4)	−21.0*
Cortical thickness	70.4 (18.9)	61.1 (24.4)	−9.4	50.8 (14.9)	−19.6*
Trabecular numbers	106.8 (14.6)	102.4 (22.7)	−4.4	92.4 (16.9)	−14.4*
Trabecular thickness	94.9 (13.1)	92.0 (17.3)	−2.8	87.1 (13.5)	−7.8*
Trabecular separation	95.8 (15.8)	105.3 (32.2)	+ 9.5	116.8 (28.1)	+ 20.9*
Tibia (normalized to age- and sex-specific reference values)	%	%	Δ %	%	Δ %
Total bone area	112.1 (17.5)	115.5 (21.3)	+ 3.4	109.8 (19.5)	−2.3
Cortical area	90.7 (15.2)	91.6 (18.5)	+ 0.9	81.7 (15.7)	−9.0
Trabecular area	98.3 (15.4)	99.4 (22.6)	+ 1.1	95.6 (20.7)	−2.6
Total vBMD	90.7 (15.2)	91.6 (18.4)	+ 0.9	81.7 (15.7)	−9.0
Cortical vBMD	85.8 (8.8)	86.4 (8.9)	+ 0.6	82.7 (7.9)	−3.1
Trabecular vBMD	98.5 (18.9)	98.5 (23.6)	−0.0	87.8 (18.2)	−10.6
Cortical thickness	67.7 (21.5)	68.9 (26.0)	+ 1.2	58.8 (23.8)	−8.8
Trabecular numbers	110.1 (18.5)	105.8 (25.4)	−4.3	102.9 (22.1)	−7.1
Trabecular thickness	90.6 (15.7)	93.2 (12.4)	+ 2.6	87.0 (19.1)	−3.6
Trabecular separation	94.1 (19.6)	101.5 (29.6)	+ 7.4	104.9 (28.9)	+ 10.8

*Abbreviations*: HR-pQCT, peripheral quantitative computed tomography; vBMD, volumetric bone mineral density

The data are normalized to age- and sex-specific reference values. The differences (∆) that are shown are relative to the non-fracture group.

* = Statistically significant (*p* < 0.05) differences to the non-fracture group

### Vitamin D status

**Fig. 2:**
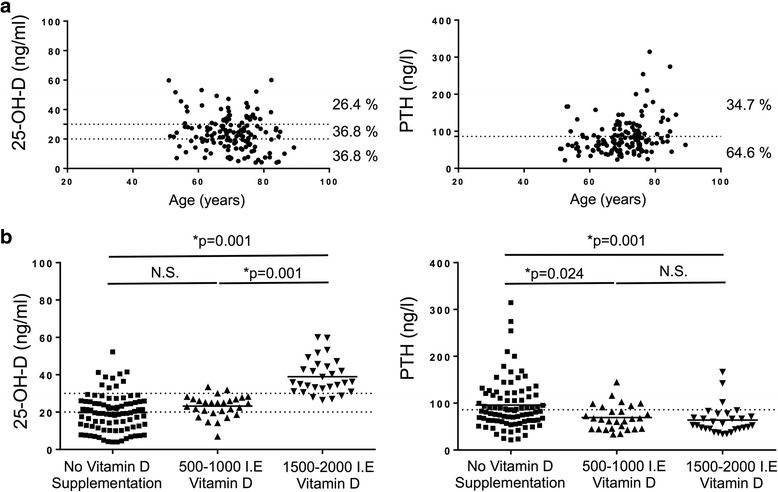
**a** Prevalence of 25-OH-D deficiency and secondary hyperparathyroidism in 144 patients scheduled for spine surgery. **b** Levels of 25-OH-D (ng/ml) and parathyroid hormone (ng/l) in patients in dependence of daily vitamin D intake. * *p* < 0.05

25-OH-D and PTH were measured in all patients. Vitamin D inadequacy was defined as a 25-OH-D level below 30 ng/ml and deficiency as levels less than 20 ng/ml. The mean 25-OH-D concentration was 24.3 ng/ml. Values ranged from 4 to 60 ng/ml. The overall prevalence of vitamin D inadequacy was 73.6% and that of deficiency was 36.8% (Fig. 2a). Severe deficiency (25-OH-D < 10 ng/ml) was present in 17 patients (11.6%). 50 (35.4%) patients showed elevated PTH levels. While 12 of these patients showed hypocalcemic hyperparathyroidism, 38 patients displayed elevated PTH levels with normocalcemia. One patient showed elevated calcium and PTH levels and was consequently diagnosed with primary hyperparathyroidism and were treated surgically with parathyroidectomy after preoperative localization of parathyroid adenoma. 44 (30.6%) patients used proton pump inhibitors and 20 (13.9%) of these patient showed elevated levels of gastrin suggesting PPI induced hypochlorhydria.

### Anti-osteoporotic treatment

57 patients used vitamin D supplementation. However, only 28 of these patients had 25-OH-D levels above 30 ng/ml suggesting adequate vitamin D supplementation (Table 4). Patients with vitamin D supplementation were divided into two groups by daily intake of vitamin D. While patients with 500–1000 I.E. of vitamin D intake per week showed mainly vitamin D inadequacy, patients with higher intake mostly showed adequate levels (Fig. 2b). However, PTH levels did not differ significantly between these groups. Patients with no vitamin D supplementation had significantly higher level of PTH (Fig. 2b). While only 16 patients (11.1%) were previously treated with specific anti-osteoporotic medication, 54 patients (37.5%) had not received specific anti-osteoporotic treatment as recommended by the evidence-based (S3) guidelines of German Association of Osteology (Table 4) despite having an indication.

**Table 4:** Anti-osteoporotic treatment in patients undergoing spinal surgery prior and recommendation after evaluation of bone mineral status

	Prior to Evaluation	Recommendation
	n (%)	n (%)
Basic therapy		
Vitamin D supplementation	57 (39.6%)	144 (100%)
Adequate vitamin D supplementation (25-OH-D within recommended range)	28 (19.4%)	
Calcium supplementation	31 (21.5%)	20 (13.9%)
Specific anti-osteoporotic treatment	16 (11.1%)	70 (48.6%)
Bisphosphonate (oral)	8 (5.6%)	26 (18%)
Bisphosphonate (intravenous)	0 (0%)	14 (9.7%)
Denosumab	8 (5.6%)	25 (17.3%)
Teriparatide	0 (0%)	5 (3.5%)
Indication for specific treatment without therapy	54 (37.5%)	

### Recommendation for anti-osteoporotic treatment

**Fig. 3:**
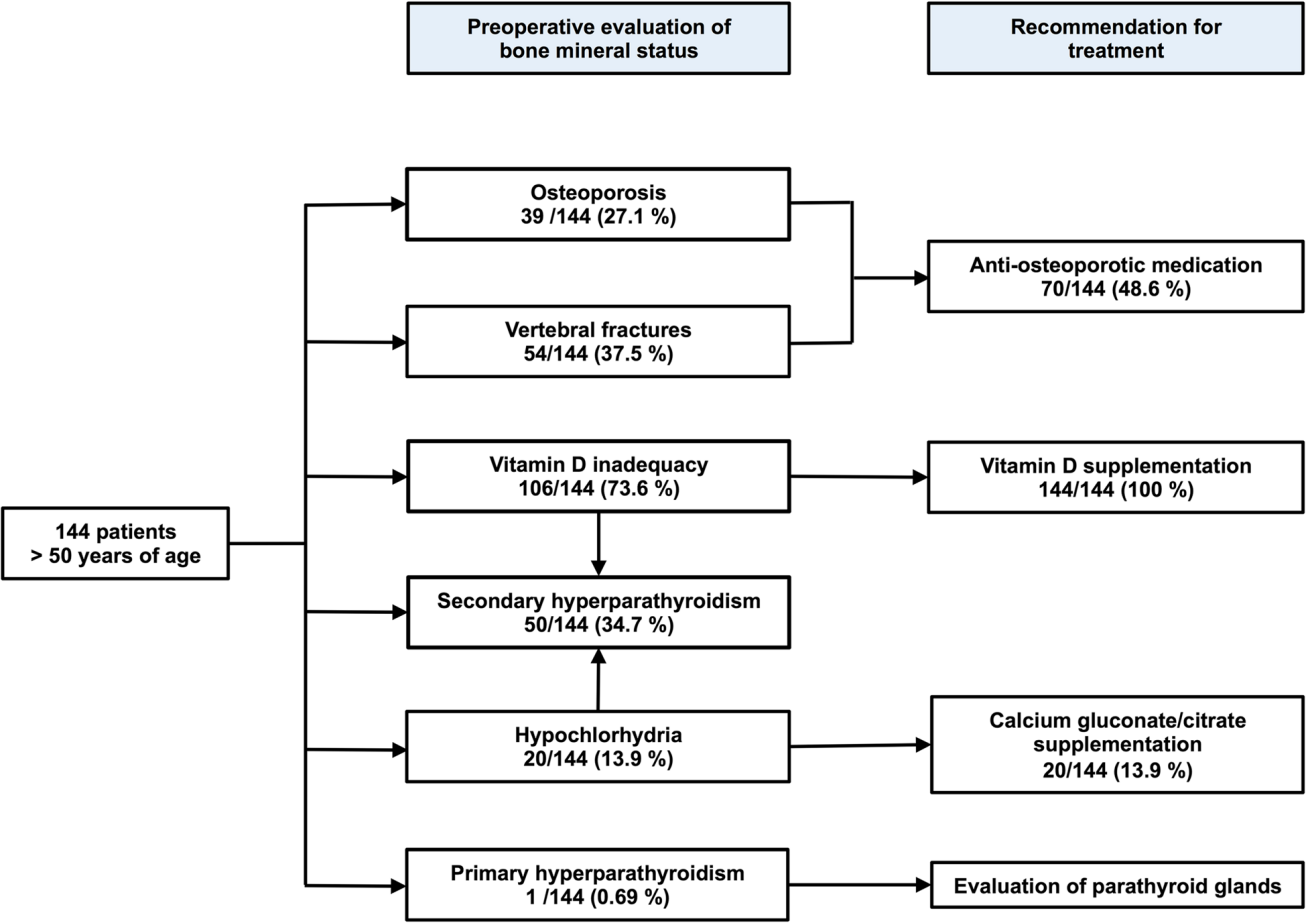
Flow chart of bone mineral status in 144 patients scheduled for spine surgery and recommendation for medical treatment

Figure 3 summarizes the major findings and implication for anti-osteoporotic treatment in the patient cohort. Vitamin D supplementation was suggested in dependence of 25-OH-D levels. Patients with hypochlorhydria due to PPI use were recommended to use calcium gluconate supplementation to improve calcium absorption [21, 22]. Indication for specific anti-osteoporotic treatment was established by using the current official recommendation of the German Association of Osteology. The choice of medication was based on an individualized decision taking risk factors and current bone mineral status and turnover into account.

## Discussion

In this study we analysed the bone health of patients requiring spinal surgery. We found a high prevalence of untreated osteoporosis, vitamin D deficiency and parathyroid hormone elevations arising from vitamin D deficiency.

Many studies suggest that BMD is one of the main factors related to poor surgical outcome of patients undergoing spine fusion operations with instrumentation [23]. The ability of screws to resist pullout from bone is directly related to BMD [24]. Moreover, longer fusion constructs are recommended for patients with an osteoporotic spine resulting in longer operative time and increased postoperative time to recovery [25]. Hence, assessment of bone quality prior to operation is crucial finding the optimal surgical strategy to proactively plan for best surgical outcomes.

According to World Health Organization (WHO) recommendations every postmenopausal women and patients with high number of osteoporosis risk factors should undergo bone mineral density screening [26]. Prevalence of osteopenia and osteoporosis for patients requiring spine surgery have been shown to be high with 46% and 31% for patients older than 50 years [27], respectively. While DXA is the gold standard to measure aBMD, it has been frequently reported that fractures often occur in patients with a T-score above − 2.5 [6] and that women with osteopenia and a prevalent fracture have the same fracture risk as women with osteoporosis [28]. In this context, it has been shown that other factors such as vitamin D deficiency or a predominant cortical bone loss are independently associated with increased fracture risk [29, 30]. Hence, to prevent future fractures and to archive better surgical outcome, it is important to identify and treat patients with reduced bone quality. It would be desirable that in elderly patients (> 50 years) scheduled to undergo elective spine surgery, bone mineral status including DXA measurement and biochemical assessment for reversible risk factors should be determined routinely and appropriate medical treatment including vitamin D supplementation initiated if necessary. In patients with a previous fracture and osteopenia or T-score within the normal range, assessment of peripheral bone structure by HR-pQCT can give useful additional information about the bone quality.

In this study, we found that patients with vertebral deformities had strong deficits in the cortical and trabecular compartments of the distal radius compared to patients without vertebral deformities. We also observed that these deficits at the radius were particularly pronounced in patients with severe vertebral deformity. Thus, the bone microarchitecture at the distal radius seems to reflect vertebral fragility better than the bone architecture at the distal tibia. Yet, it is important to mention that in our study the overall peripheral bone microstructure was only assessed in patients with a T-score below − 1.5. Thus, the patients without vertebral deformities in this study were also in the range of osteopenia or osteoporosis regarding DXA T-score. There are only a few clinical studies that have compared patients with and without vertebral fractures in terms of peripheral changes in the trabecular and cortical compartments. These studies have reported conflicting results in differences observed only at the radius (31), or at the radius and tibia [31–33]. However, studies that reported similar differences at the radius and the tibia compared healthy control patients to osteopenic or osteoporotic patients with vertebral deformities [31, 34]. Furthermore, studies on patients with various kinds of fragility fractures support the notion that deterioration in bone microstructure plays an important role in the pathogenesis of fractures in general, and that such bone alterations are particularly pronounced at the distal radius [35, 36]. This may be partially explained be the protective effect of weight-bearing on bone microstructure [8, 37]. Moreover, both primary and secondary hyperparathyroidism have been associated with a predominant deterioration of bone microstructure at the distal radius [38, 39]. The possibility that peripheral bone structure can predict the severity of vertebral fracture is of high clinical interest because severe fracture cases have a higher risk of developing changes in spine structure such as kyphotic deformities and spinal stenosis due to sagittal imbalance and degenerative changes. These degenerative alterations falsify aBMD measurement at the spine. Moreover, patients with spinal fusion and/or spinal degeneration in combination with hip joint replacement are not measureable with DXA. In these patients the assessment of the peripheral bone structure at the distal radius could be a useful tool to evaluate and monitor skeletal fragility.

Vitamin D plays an essential role in bone remodelling and is crucial for adequate uptake of calcium. Vitamin D deficiency has been identified as an important risk factor for stress fractures in several studies [15, 40]. Correcting vitamin D deficiency can significantly increase bone mass in elderly people [41]. Nevertheless, a number of studies have reported high prevalence of deficient or inadequate vitamin D levels in patients requiring spinal surgery. Stoker et al. found that nearly 85% of adult patients undergoing spinal fusion showed deficient or inadequate 25-OH-D levels in the United States [20]. This is an even higher rate of insufficiency than in our study with 73.6% of patients showing inadequate vitamin D status. Other studies report similarly rates of vitamin D insufficiency comparable to the vitamin D status of the general population [16, 42].

Vitamin D deficiency often result in secondary hyperparathyroidism causing increased bone resorption. In our study 35.4% of patients showed elevated PTH levels. Some studies suggest that levels of 20 ng/ml are sufficient to avoid secondary hyperparathyroidism, while other studies argue for higher minimum threshold (30 ng/ml) to maintain skeletal health [43]. In our study, patients who received more than 1500–2000 I.E. vitamin D per day mostly reached levels of 25-OH-D > 30 ng/ml, while patients with lower intake (daily 1000 I.E.) showed levels between 20 and 30 ng/ml. However, PTH did not differ between these groups suggesting that levels above 20 ng/ml are generally enough to avoid secondary hyperparathyroidism. Nevertheless, we think based on the current literature and own previous studies it is reasonable to recommend higher intake to reach 25-OH-D levels above 30 ng/ml for optimal osteoid mineralization [43]. Most importantly, with proper treatment patients can have secondary hyperparathyroidism reversed in relatively short time before undergoing elective spinal surgery.

As gastric calcium solubility has been shown to be pH-dependent, hypochlorhydria which is defined by reduced stomach acid production can induce calcium malabsorption which negatively affects bone mineralization [21]. Therefore, patients with hypochlorhydria which can be caused by long term use of proton pump inhibitors (PPI) display higher prevalence for osteoporosis and increased fracture risk [44]. As calcium gluconate or citrate have been shown to be very effective in correcting calcium malabsorption in patients with reduced stomach acid production [21], we recommend calcium gluconate or citrate supplementation in patients with permanent use of PPI.

Prevention is the most important principle in the management of osteoporosis. This is especially important for patient with previous fragility fractures. However, recent studies suggest that there is a large gap between patients with fragility fractures and those receiving appropriate anti-osteoporotic therapy [45]. While the availability of acute surgical treatment for vertebral fractures is considered excellent in Germany, many studies show a deficient in postoperative medical management of patients underlying low BMD resulting in high prevalence of untreated osteoporosis ranging from 30 to 70% [46, 47]. In our study, we found that 37.5% of patients had not received anti-osteoporotic treatment despite having an indication.

Our study has several limitations due to its retrospective design. Firstly, it has to be taken into account that the mean age in our study group was 70.1 years, an age where prevalence of osteoporosis is dramatically increasing. In fact the prevalence of osteoporosis in women > 70 years-old is 13–26% according to prior studies [48]. Secondly, compression fractures of the spine composed a large portion of patients which elevates the prevalence of osteoporosis and increases the number of patients in need of specific anti-osteoporotic therapy. Thirdly, regarding vitamin D status geographic differences has to be considered as northern European population generally has a higher risk to develop vitamin D deficiency and secondary hyperparathyroidism due to reduced sun light exposure.

## Conclusion

This study shows that despite recent advances in the diagnosis and treatment of osteoporosis, elderly people scheduled for spinal surgery often show diminished bone mineral density, inadequate vitamin D status, elevated PTH levels and frequently have no anti-osteoporotic therapy. Future studies should aim to investigate the outcome of spinal surgery with regard to the preoperative bone mineral status and the effects of accurately timed preoperative anti-osteoporotic treatment. Considering the potential negative effects of low BMD and vitamin D deficiency for surgical outcome, preoperative screening and initiation of therapy for elderly patients is highly recommended.

## Abbreviations

25-OH-D: 25-hydroxyvitamin D
ALP: Alkaline phosphatase
BAP: Bone specific alkaline phosphatase
BMD: Bone mineral density
DPD: Deoxypyridinoline
DXA: Dual-energy X-ray absorptiometry
HR-pQCT: High-resolution peripheral quantitative computed tomography
PTH: Parathyroid hormone
WHO: World Health Organization
